# *Granulicatella adiacens* Subacute Bacterial Endocarditis Presenting as Diffuse Alveolar Hemorrhage and Infection-Related Glomerulonephritis

**DOI:** 10.1155/2022/5565906

**Published:** 2022-03-07

**Authors:** Kevin Dao, Pooja Patel, Kunjan Udani, Erin Pollock, Maryam Gondal

**Affiliations:** ^1^Department of Internal Medicine, Mercer University School of Medicine—Grand Strand Medical Center, Myrtle Beach, SC, USA; ^2^Department of Nephrology, Mercer University School of Medicine—Grand Strand Medical Center, Myrtle Beach, SC, USA

## Abstract

We present a case of a 69-year-old male with a past medical history of prostate cancer, chronic mitral valve regurgitation, and recent dental cleaning who presented to the hospital with shortness of breath, anemia, and acute renal failure. Due to unexplained creatinine rise, a renal biopsy was obtained which was suspicious for infection-related glomerulonephritis (IRGN). Further workup confirmed subacute endocarditis according to modified Duke's criteria. The patient's blood culture became positive for *Granulicatella adiacens*, a nutritionally variant streptococcus. The patient later developed acute respiratory failure from diffuse alveolar hemorrhage (DAH). Subacute infective endocarditis can result in serious morbidity and mortality due to its insidious symptoms and subsequent fatal complications.

## 1. Introduction

Subacute bacterial endocarditis (SBE) is a fatal infection that warrants prompt evaluation, treatment with antibiotics, and surgical intervention in severe cases. The symptoms can be variable, with most patients presenting with nonspecific symptoms such as fever, chills, anorexia, weight loss, and dyspnea, progressively worsening over weeks to months [1]. Subacute bacterial endocarditis usually precipitates in the setting of prior valvular diseases and its risk increases with ages greater than 60 years, history of intravenous (IV) drug use, rheumatic heart fever, prosthetic heart valves, poor dentition, and recent dental procedure [2]. It has an estimated incidence of 12.7 per 100,000 people in the United States annually, most commonly due to *Streptococcus viridans* [2]. Here, we present a case of *Granulicatella adiacens* subacute bacterial endocarditis diagnosed after the workup of unknown glomerulonephritis later complicated by DAH.

## 2. Case

A 69-year-old male with a history of prostate cancer status after radical prostatectomy, currently undergoing radiation therapy, and chronic moderate mitral valve regurgitation (MVR) presented with progressive dyspnea on exertion and weight loss for the past five weeks. Approximately eight weeks before presentation, the patient reported undergoing dental cleaning and was found to have a small mandibular abscess a few weeks after. The patient did not receive antibiotic prophylaxis prior to dental cleaning; however, antibiotics were given if it were to worsen. Unfortunately, the patient did not take the antibiotics and began to have low grade fevers, chills, fatigue, weight loss, and worsening shortness of breath which was initially contibuted to his radiation treatment. The patient's symptoms did not improve so he decided to go to the emergency room for further evaluation.

**Figure 1:**
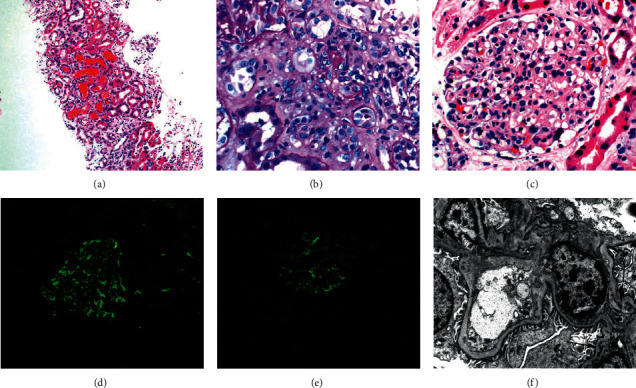
Renal biopsy demonstrating mild proliferative and exudative glomerulonephritis with IgM-dominant deposition consistent with infection-related glomerulonephritis. (a) Acute tubular injury with red blood cell casts. (b) Arteriolar hyalinosis. (c) Proliferative glomerulonephritis with neutrophils. (d) IgM immune complex deposition. (e) C3 immune complex deposition. (f) Immune complex mesangial deposits on electron microscopy.

**Figure 2:**
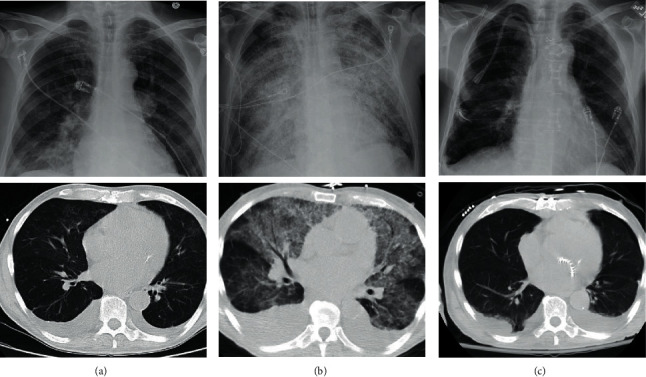
Chest imaging: chest X-ray and chest CT of progression of diffuse alveolar hemorrhage. (a) Day 5 shows right basilar opacity. (b) Day 10 shows diffuse alveolar infiltrates consistent with DAH. (c) Day 15, which was approximately five days postoperation, shows resolution of alveolar hemorrhage.

On admission, the patient was afebrile and tachycardic with a heart rate of 102 beats per minute. Physical examination was relevant for III/VI blowing systolic murmur at the apex radiating to the axilla. The remainder of the physical exam was unremarkable. The patient was found to have microcytic anemia, acute decompensated heart failure, and acute renal failure. Laboratory workup revealed a normal white blood cell count with predominant neutrophils (84.3%), hemoglobin (Hgb) of 8.9 gm/dL (11.6–15.4 gm/dL), and mean corpuscular value (MCV) of 84.8 fL (79.2–97.2 fL). Peripheral blood smear was consistent with microcytic anemia with no evidence of schistocytes. His blood urea nitrogen (BUN) was 106 mg/dL (7–20 mg/dL) and creatinine (Cr) was 5.58 mg/dL (Cr 0.7–1.5 mg/dL). Urinalysis was significant for proteinuria of 20 mg/dL (0 mg/dL), hematuria greater than 1.0 mg/dL (0 mg/dL), RBC/HPF greater than 100 (0-1 RBC/HPF), and urine protein-to-creatinine ratio of 0.4 mg/mg (less than 0.2 mg/g). Inflammatory markers showed C-reactive protein (CRP) of 4.4 mg/L (8–10 mg/L) and erythrocyte sedimentation rate of 44 mm/hr (0–30 mm/hour). The patient had hypocomplementemia with C3 less than 40 mg/dL (normal: 80–178 mg/dL) and C4 less than 10.9 (normal: 12–42 mg/dL). Other workups including serum protein electrophoresis (SPEP), urine protein electrophoresis (UPEP), perinuclear anti-neutrophilic cytoplasmic antibodies (P-ANCA), cytoplasmic anti-neutrophilic cytoplasmic antibodies (C-ANCA), and antinuclear antibodies (ANA) were unremarkable. Due to abnormal renal function and unclear etiology, a renal biopsy was performed on day 5 of admission that demonstrated infection-related glomerulonephritis (Figure 1). Blood cultures were obtained prior to antibiotics, and the patient was started on ceftriaxone for empiric coverage of subacute bacterial endocarditis. Blood cultures initially grew Gram-positive cocci resembling streptococcus. Further speciation was performed by an outside laboratory using matrix-assisted laser desorption ionization-time-of-flight mass spectrometry (MALDI-TOF MS). On day 9 of hospitalization, final blood cultures were identified as *Granulicatella adiacens.* Sensitivities showed resistance to cefepime and ceftriaxone but sensitive to erythromycin, penicillin, and vancomycin (Table 1). The patient was transitioned to ampicillin and gentamicin which is the treatment of choice in the setting of a nutritionally variant streptococcus and high risk of treatment failure and mortality. Transthoracic echocardiogram (TTE) revealed severe mitral regurgitation, moderate prolapse involving posterior leaflet, and presence of papillary, echogenic, highly mobile vegetation on mitral valve (MV). TEE later confirmed vegetation and suspicion of subacute bacterial endocarditis. Cardiac catheterization for preoperative surgical evaluation demonstrated moderate obstructive multivessel disease. Cardiothoracic surgery was consulted and recommended MV replacement and coronary artery bypass surgery (CABG). On day 10, the patient developed hemoptysis, worsening anemia, and dyspnea. Computed tomography (CT) of the chest was obtained (Figure 2(b)) and showed new diffuse bilateral ground glass opacities in lungs with right larger than left pleural effusions. Mechanical ventilation support was required due to hypoxia, and urgent bronchoscopy confirmed the diagnosis of DAH due to massive hemoptysis despite sequential bronchoalveolar lavage. On day 11, the patient had successful mitral valve replacement and CABGx3. He was started on epoprostenol for severe pulmonary hypertension of 70 mmHg (normal: 8–20 mmHg), continuous renal replacement therapy (CRRT), and inotropic and vasopressor support. On hospital day 13, the patient continued to improve and was extubated. Vasopressor and inotropic medications were weaned off. CRRT was discontinued and transitioned to intermittent hemodialysis. On hospital day 15, the patient had resolution in dyspnea, kidney function (Cr 0.8 mg/dL), and alveolar hemorrhage (Figure 2(c)).

On hospital day 18, he developed a small bowel obstruction requiring an ileocecectomy. The patient later became septic, likely secondary to suspected abdominal source requiring broad spectrum antimicrobials to include cefepime, metronidazole, and micafungin. The patient improved and was preparing for discharge to rehab; however, he later developed acute hypoxic respiratory failure and cardiogenic shock, requiring intubation, and vasopressor support. Ultimately, he succumbed to pulseless electrical activity arrest driven by hypoxia.

## 3. Discussion

Bacterial endocarditis is a multisystem disease categorized into two subtypes, acute endocarditis, with a rapid and life-threatening course, and subacute endocarditis, with a gradual disease progression over weeks to months [1]. Pathogens have multiple potential entry points into the bloodstream but usually enter through endothelial injury. Once the organism enters the bloodstream, it can attach to an abnormal cardiac surface, bury itself within a protective matrix, and begin to proliferate. The resultant vegetation created can detach and embolize, leading to visceral infarcts and systemic infection [3]. Potential etiologies of endocarditis include Streptococci, *Staphylococcus* such as *Staphylococcus aureus*, Enterococci, and HACEK organisms (*Haemophilus*, *Aggregatibacter*, *Cardiobacterium*, *Eikenella*, and *Kingella*) [1]. Mortality within 30 days is reported to be 10–24%, and one-year mortality is slightly higher (22–37%) [4]. Subacute bacterial endocarditis is a difficult diagnosis and clinicians should be suspicious if patients with high risk factors for endocarditis present with nonspecific symptoms. Our patient was first evaluated for subacute bacterial endocarditis when exploring possible etiologies for acute renal insufficiency, and a renal biopsy was obtained confirming infection-related glomerulonephritis.

Acute renal failure is one of the most common initial findings of bacterial endocarditis, and its association with glomerular lesions has been documented for over 100 years [5]. Approximately 50–60% of patients with infective endocarditis (IE) will have acute kidney injury and about 20% of patients can develop glomerulonephritis [6–8]. In a large biopsy-based IE associated GN study, Boils et al. revealed that over half the patients had no prior cardiac issues and most cases were associated with the tricuspid valve secondary to *Staphylococcus* [5]. Low complement levels were seen in 56% of patients, and ANCA were present in 28% [5]. Renal biopsies showed a higher propensity to immunoglobulin depositions and a necrotizing crescentic GN pattern [5]. IRGN can have glomerulosclerosis, tubular atrophy, interstitial fibrosis, arteriosclerosis, and arteriolar hyalinosis on renal biopsies. Glomerular injury can occur through subendothelial and mesangial deposits with antigen-antibody immune complexes that activate immune response pathways. Light microscopy should show diffuse glomerular neutrophil infiltrates, and immunofluorescence should show a C3 predominant granular mesangial and glomerular wall staining resembling “a starry sky pattern.” Codeposition can happen with one or other immune reactants such as IgG, IgM, and IgA; however, IgG is typically more frequent. Electron microscopy can also show electron dense mesangial and sub-endothelial deposits [9]. Although appropriate treatment for renal impairment from IE has not been established, most experts have agreed on antibiotic therapy, surgery, and consideration for dialysis and immunosuppressive therapy [10].

Diffuse alveolar hemorrhage is a rare complication associated with infective endocarditis. Etiologies such as vasculitis, autoimmune diseases, infection, and valvular pathology have been depicted; however, acute mitral valve regurgitation is rarely reported [11, 12]. Our patient had an acute on chronic mitral valve regurgitation secondary to endocarditis and developed subtle signs of right-sided alveolar hemorrhage. Patients with these features are typically misdiagnosed as having a right lower lobe pneumonia (Figure 2(a)) and quickly develop diffuse alveolar hemorrhage (Figure 2(b)). Patients with this sign have an increased risk of mortality from delay of diagnosis. Patients usually have right-sided involvement greater than left due to the mitral valve's regurgitation jet flow, which is directed towards the upper right pulmonary vein causing back flow to the right side of the heart [13]. The left atrium does not have time to compensate for the acute pressure and will cause progressive dyspnea and hemoptysis. Mitral valve regurgitation and a right-sided infiltrate should be quickly evaluated for DAH, intubated for airway protection, and transferred to the intensive care unit for further management and evaluation for urgent valvular repair.

*Streptococcus viridans* is implicated in 50–80% of subacute bacterial endocarditis cases although *Staphylococcus epidermidis* is seen in approximately 20–30% of cases [14]*. Staphylococcus aureus* is the most common organism in acute bacterial endocarditis [2]. Blood culture negative endocarditis may be found in 5–10% of endocarditis raising the suspicion for slow growing organisms such as *Granulicatella adiacens* [15]. *Granulicatella adiacens* can be found in approximately 5% of all cases of IE. This is a nutritionally variant Streptococci-like species that is facultatively anaerobic. It has difficulty growing in standard blood cultures requiring pyridoxal and L-cysteine growth factors, which are not usually found in standard sheep blood agar (SBA) [16, 17]. In our case, cultures had to be sent to an outside facility for further speciation. *Granulicatella* adiacens is more resistant to antibiotics than its Streptococci cousins but is known to be sensitive to clindamycin, rifampicin, erythromycin, and vancomycin. *G. adiacens* belongs to the normal flora within the mouth and intestinal tract but has been associated with IE after entering the bloodstream. These organisms seem to have a higher affinity towards binding cardiac valves, most commonly mitral and aortic valves. The treatment of choice is an antibiotic combination with penicillin and aminoglycoside; however, up to 41% of patients experience treatment failure and up to 27% will require valve replacement [15–17].

Bacterial endocarditis can mask itself within many different disease processes. It is an embolic disease with often devastating implications. Every organ could potentially be affected, and physicians should maintain a high suspicion for IE in patients with known risk factors presenting with nonspecific symptoms.

## 4. Conclusion

Due to its potential to cause life-threatening complications such as diffuse pulmonary hemorrhage and infection-related glomerulonephritis, it is imperative to diagnose subacute bacterial endocarditis promptly and implement appropriate management to improve outcomes.

## Figures and Tables

**Table 1:** Sensitivities of *Granulicatella adiacens*.

Antibiotic	MIC	Interpretation
Cefepime	8	R
Ceftriaxone	>2	R
Chloramphenicol	4	S
Clindamycin	0.500	I
Erythromycin	≤0.250	S
Penicillin	0.060	S
Vancomycin	1	S

MIC: minimum inhibitory concentration (in *μ*g/mL); R: resistant; I: indeterminate; S: sensitive.

## Data Availability

The data that support the findings of this study are available within the article.

## Additional Points

*Learning Objective*. Recognize diffuse alveolar hemorrhage and infection-related glomerulonephritis as potential complications of subacute bacterial endocarditis.
